# Correction to ‘Relationship between maternal body composition changes and heavy for date infants in pregnant women with diabetes’

**DOI:** 10.1111/jdi.70475

**Published:** 2026-10-27

Eto E, Kato M, Kirino S, et al. Relationship between maternal body composition changes and heavy for date infants in pregnant women with diabetes. *J Diabetes Investig*. 2025;16:1910–1918. https://doi.org/10.1111/jdi.70131.

In the ‘Funding’ section, the sentence ‘This study received no specific grants from any funding agency, commercial sector, or not‐for‐profit sector’. was incorrect. This should have read:

The article publication charge was supported by the “Initiative for Realizing Diversity in the Research Environment” from MEXT, Japan, through Okayama University.

We apologize for this error.

